# The Utility of High-Flow Nasal Oxygen Therapy in a Patient with Pneumonia Undergoing Lower Limb Orthopaedic Surgery: A Case Report

**DOI:** 10.5152/TJAR.2022.21250

**Published:** 2022-12-01

**Authors:** Neeraj Kumar, Pavan Kumar Kandrakonda, Mithun Rathinasamy, Abhyuday Kumar, Kunal Singh, Anup Kumar

**Affiliations:** 1 Department of Trauma and Emergency, All India Institute of Medical Sciences Patna, Bihar, India; 2 Department of Anaesthesiology, All India Institute of Medical Sciences Patna, Bihar, India; 3 Department of Orthopaedics, All India Institute of Medical Sciences Patna, Bihar, India

**Keywords:** Airway management, geriatric patient, high flow oxygen cannula, intraoperative period, pneumonia, regional anaesthesia

## Abstract

A high-flow nasal cannula is commonly used to manage hypoxic respiratory failure. High-flow nasal cannula not only reduces respiratory effort and work of breathing but also provides better patient comfort. It lowers mortality compared to standard oxygen therapy or non-invasive ventilation and is associated with more ventilator-free days. A 60-year-old female presented for the correction of ankle fracture with pneumonia and successfully underwent lower limb orthopaedic surgery under the subarachnoid block with the use of high-flow nasal oxygen therapy. High-flow nasal cannula may be considered as one of the useful options for intraoperative management of pneumonia in patients undergoing lower limb surgery under regional anaesthesia.

## Introduction

A high-flow nasal cannula (HFNC) is commonly used to manage hypoxic respiratory failure. It is associated with more ventilator-free days and lower mortality as compared to standard oxygen therapy or non-invasive ventilation.^1^ The heated and humidified oxygen allows the delivery of gases at temperatures between 33°C and 43°C and 95%-100% humidity via nasal cannula at flow rates ranging from 20 to 60 L min^−1^.^2^ It also provides positive end-expiratory pressure (PEEP), nearly to a value of 1 cm of H_2_O for every 10 L min^−1^ flow.^3^ High-flow nasal cannula fills the anatomical dead space with oxygen and decreases the rise of the partial pressure of carbon dioxide (PaCO_2_). This technique not only reduces respiratory effort and work of breathing but also provides better patient comfort. Patients with injuries or fractures to limbs, especially lower limbs, were not able to ambulate. This prolonged immobilisation may lead to decreased ventilation, atelectasis, and pneumonia. The risks of respiratory complications are always more in geriatric age groups due to progressive increases in physical frailty, osteopenia, and osteoporotic changes in long bones.

## Case Presentation

**Figure 1.**
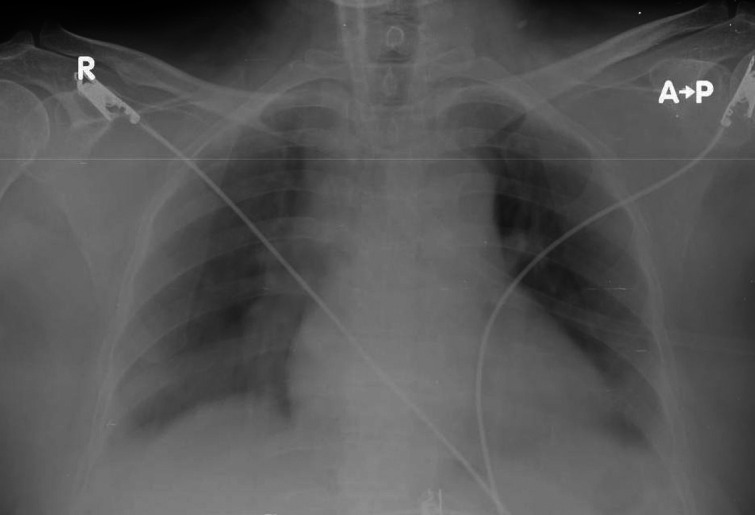
Preoperative chest x-ray showing features of pneumonia.

**Figure 2.**
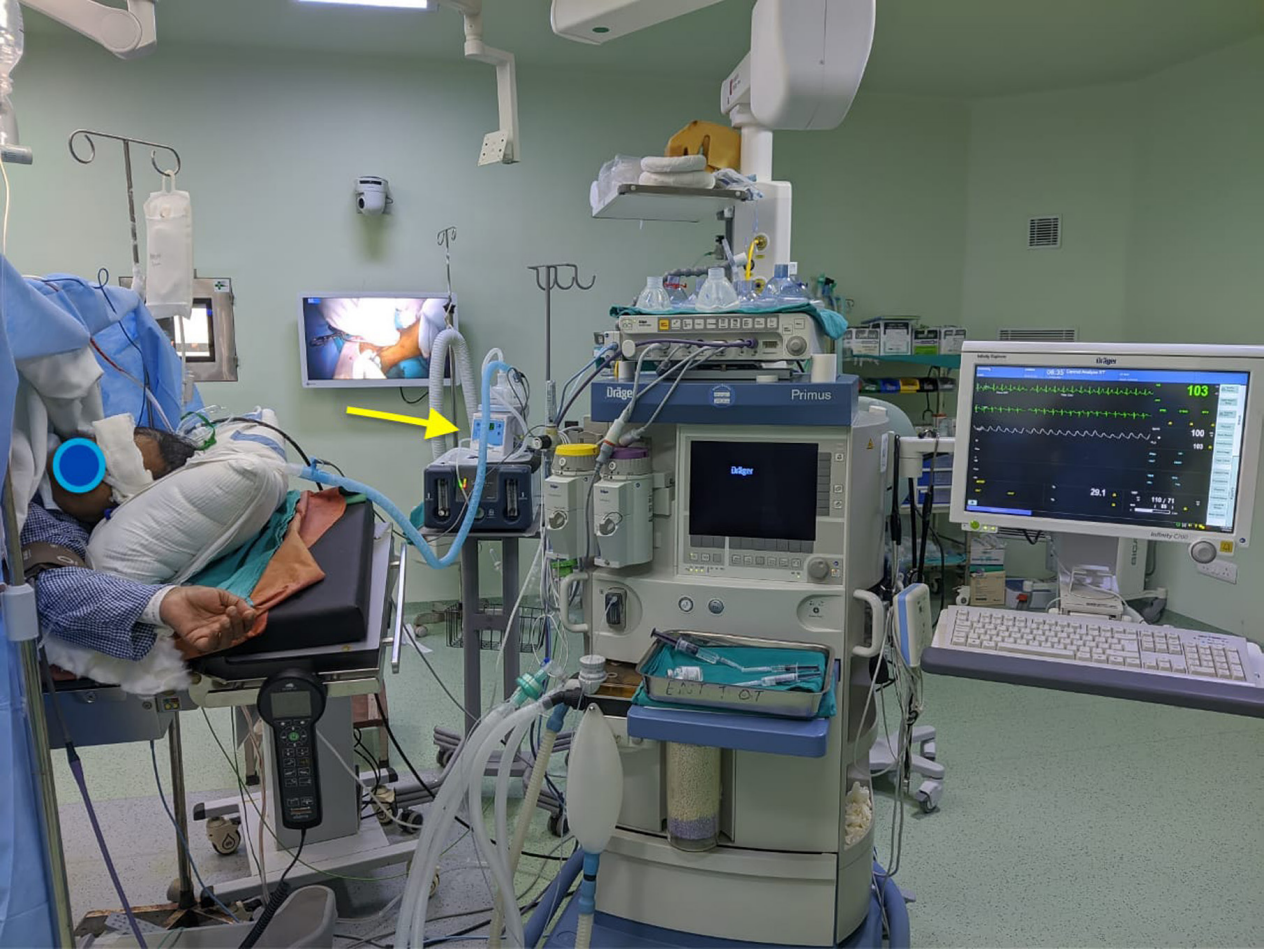
Arrow showing intraoperative use of high-flow nasal cannula in patient undergoing ankle surgery under subarachnoid block.

**Figure 3.**
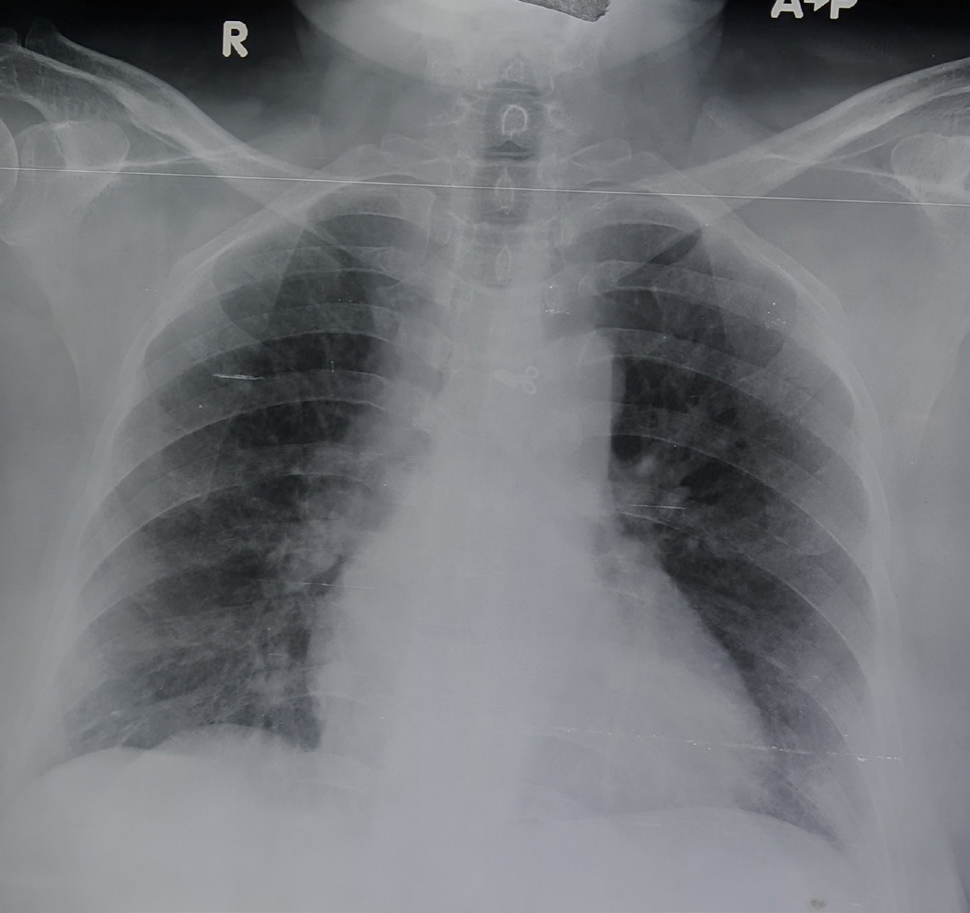
Chest X-ray (AP View) during discharge.

Here, we report a case of ankle fracture with pneumonia who successfully underwent lower limb orthopaedic surgery under the subarachnoid block with the use of high-flow nasal oxygen therapy. The consent to publish this report was obtained from the patient. A 60-year-old female with 78 kg weight presented to the orthopaedics outpatient department with pain and swelling in her right ankle joint for 20 days. She had a history of twisting injuries to the ankle following a fall on the floor. The swelling was localised to the ankle joint, and the pain was radiating to the heel and was aggravated on standing. She was having a cough and shortness of breath for the last 3 days. She had no history of any co-existing co-morbidities. The vitals at the time of admission was heart rate (HR) 110 bpm, respiratory rate (RR) 28 per minute, and blood pressure (BP) 130/80 mm Hg, and on chest auscultation, bilateral coarse crackles, rales, and diminished air entry were heard at the lung bases. The chest X-ray was suggestive of lobar pneumonia, so a diagnosis of community-acquired pneumonia was made (Figure 1). As per our COVID-19 institutional protocol, she was admitted to the holding area, and a nasopharyngeal sample for reverse transcriptase-polymerase chain reaction was sent, which was tested negative. The patient was admitted to our intensive care unit and the baseline arterial blood gas (ABG) was pH: 7.40, PaO_2_: 66 mm Hg, PaCO_2_: 28 mm Hg, HCO_3_: 21 meq L^−1^, and lactate: 1.3 mmol L^−1^. She was kept on continuous positive airway pressure (CPAP) mode with PS/PEEP 9 cm H_2_O and FiO_2_ of 50%. The orthopaedics team decided to correct the fracture as soon as possible. All her baseline biochemical parameters were normal except for raised total leucocyte count (TLC) count, which was 13 × 10^9^ mm^−^^3^. A broad-spectrum antibiotic, inhalational steroids such as budesonide, and pantoprazole were advised. We planned for subarachnoid block (SAB) and intraoperative use of HFNC. On the morning of surgery, the patient’s baseline oxygen saturation was between 86% and 88%, with a respiratory rate of 30 rate min^−1^. On chest auscultation, bilateral basal coarse crackles were still present. After obtaining written informed consent and applying all standard ASA monitors, the SAB was performed at L_3_-L_4_ space. She was placed in a left lateral position and following local skin infiltration, a 25G Quincke spinal needle (Vygon India Pvt.Ltd., Gurgaon, India) was used to perform SAB after assessing the free flow of clear cerebrospinal fluid (CSF). Then, 2 mL of 0.5% hyperbaric bupivacaine (10 mg) was injected into the subarachnoidal space. High-flow nasal cannula (SAANS PRO, InnAccel Technologies Private Limited, Karnataka, India) was applied with a flow rate of 30 L min^−1^ and a FiO_2_ of 0.6 (Figure 2). The intraoperative vital signs remained stable throughout the intraoperative period with oxygen saturation of >96%. The intraoperative ABG were pH: 7.39, PaO_2_: 94 mm Hg, PaCO_2_: 32 mm Hg, HCO_3_: 22 meq L^−1^, and lactate: 0.9 mmol L^−1^. The surgery lasted for 120 minutes and the entire intraoperative period was uneventful. The patient was very comfortable and cooperative and no sedation was used. In the postoperative period, we planned to continue her on HFNC. In the postoperative period, she was kept on HFNC for another 18 hours in the same setting. Gradually, her crackles decreased, and her oxygen requirement was also reduced. At the end of the third day, she was comfortable on room air with clear auscultation of bilateral normal vesicular breath sounds in all zones, and subsequently, she was discharged from the hospital on the 10th day (Figure 3).

## Discussion

In recent times, apart from its role in acute respiratory failure, HFNC is becoming a preferred mode in managing difficult airways, improving gas exchange post-abdominal and cardiac surgery, in the post-extubation and pre-intubation period in intensive care, and facilitating bronchoscopy.

There are various methods of administering oxygen therapy, that is, via nasal cannula or face mask, for acute and chronic treatment of hypoxaemia. However, the use of a traditional cannula or face mask can provide oxygen up to 15 L min^−1^, but it provides discomfort to the patient on increasing the oxygen flow due to insufficient heating and humidification.

Non-invasive mechanical ventilation (NIV) along with conventional oxygen therapy (COT) may improve outcomes in acute life-threatening hypercapnic respiratory failure in patients with chronic obstructive lung disease.^4^ But NIV may be poorly tolerated, and frightening (due to the high pressures delivered in the airways), difficulty in synchronising breathing, claustrophobia, stomach distension, and mask-related side effects like nose sores and skin lesions over the bridge of the nose despite its effectiveness.^5^

High-flow nasal cannula is an emerging technique designed to provide oxygen at high flows via an interface consisting of a silicone cannula that fits in the nares without occlusion. This offers better comfort, compared with NIV, and more efficient oxygenation than COT.^6^

Doyle et al^7^ in their study demonstrated that pre-oxygenation and apneic oxygenation using transnasal humidified rapid insufflation ventilatory exchange (THRIVE) were associated with a lower incidence of desaturation during emergency intubation of patients, and it may provide a safe method of oxygenation to patients during intubation in the emergency operating room.

Haywood et al^8^ in their multicentric trial observed the use of HFNC as a beneficial role in the acute management of respiratory failure (pulmonary oedema) associated with acute decompensated heart failure as they create a mild positive intrathoracic pressure which may decrease the preload to the right ventricle and increase the hydrostatic pressure in the alveoli improving the cardiopulmonary status of heart failure patients.

Chung et al^9^ in their retrospective study suggested the clinical effectiveness of HFNC at a flow rate of 40-60 L min^−1^ in hypoxaemic patients during diagnostic and interventional bronchoscopy procedures.

Hernández et al^10^ in their multicentric randomised non-inferiority clinical trial on 604 adults concluded that the proportion requiring reintubation was 22.8% with (HFNC) high-flow oxygen therapy versus 19.1% with non-invasive ventilation, and post-extubation respiratory failure was observed in 26.9% with high-flow therapy versus 39.8% with non-invasive ventilation and may prove better for patients requiring reintubation with lower post-extubation respiratory failure. Regional anaesthesia is often preferred in ankle fracture surgery due to its better safety profile and postoperative pain control than general anaesthesia.^11^ High-flow nasal cannula can be a useful tool in the perioperative management of these patients with hypoxaemic respiratory failure.

This was a late-presented trimalleolar unstable and displaced fracture that needed immediate correction as per the orthopaedic surgeon's advice as the fracture was more than 2 weeks old. A further delay may lead to difficult or failed reduction with high chances of secondary osteoarthritis. So, we planned for SAB in this patient and applied HFNC in the left lateral position. We avoided peripheral nerve blocks in our case because of patient refusal and other potential problems. It takes more time to perform, delays the ability to monitor postoperative neurological function, and masks the features of acute compartment syndrome.^12^ Although sepsis is a relative contraindication for SAB, we choose it, considering the risk versus benefit of this technique in the absence of coagulopathy. In our case, SAB is preferred over general anaesthesia because of the following advantages: better postoperative analgesia, preserved spontaneous breathing leading to lesser cephalad displacement of the diaphragm and lesser risk of atelectasis, closing capacity, functional residual capacity not much affected, and pulmonary gas exchange is better maintained.^13^ We also avoided the strong stimulation of intubation or increased risk of ventilator-associated pneumonia and risks of bronchoconstriction on extubation following general anaesthesia. However, some of the disadvantages of using HFNC may be due to high nasal flow and they are nasal pain, nasal mucosa irritation, increased risks of epistaxis, and high flow can lead to gastric distension which further jeopardises respiration. But we have not noticed any such events in our case.

High-flow nasal cannula may be considered as one of the useful options for intraoperative management of pneumonia under regional anaesthesia. High-flow nasal cannula has already been used in the management of difficult airway, post-extubation and pre-intubation periods in the operation theatre, as well as intensive care. High-flow nasal cannula can be a useful tool in the perioperative management of hypoxaemic respiratory failure.
